# Solitary Plasmacytoma of the Breast: A Case of an Uncommon Breast Neoplasm

**DOI:** 10.1155/2023/9622042

**Published:** 2023-06-03

**Authors:** Sean McCormack, Eyad Hamad, Amar Hamad

**Affiliations:** ^1^Saint James School of Medicine, Cane Hall Road, Arnos Vale, Saint Vincent and the Grenadines; ^2^Northwestern School of Medicine, 10604 SW Hwy Ste 200, Chicago Ridge, IL 60415, USA; ^3^Christ Hospital, 10604 SW Hwy Ste 200, Chicago Ridge, IL 60415, USA

## Abstract

Plasmacytoma is a rare cancer that originates from a single plasma cell and is characterized by the abnormal proliferation of monoclonal plasma cells. It is typically localized in a single area of the body, most commonly in the bone or soft tissue. Solitary plasmacytoma can be further classified as either solitary plasmacytoma of bone (SPB) or solitary extramedullary plasmacytoma (SEP or EMP). Diagnosis may be delayed in symptomatically silent plasmacytomas, but early diagnosis and prompt treatment are crucial for the management of this disease. The mean age for patients with plasmacytoma varies depending on the specific type of plasmacytoma, but generally, it is more common in older adults. Soft tissue plasmacytomas are uncommon, and plasmacytomas manifesting within the breast are extremely rare, especially when they are not a manifestation of multiple myeloma (MM). This report presents a case of SEP of the breast in a 79-year-old female patient. This rare disease needs to be studied further in terms of long-term survival and disease progression to MM. By raising awareness and understanding of plasmacytoma, we aim to improve outcomes and quality of life for patients affected by this disease.

## 1. Introduction

Plasmacytoma is a rare type of cancer that originates from a single plasma cell and is typically localized in a single area of the body, most commonly in the bone but also in soft tissues. Plasmacytomas are a type of early-stage plasma cell dyscrasia/neoplasm, a group of heterogeneous disorders. Plasma cell dyscrasias are characterized by abnormal proliferation of monoclonal plasma cells. Plasmacytoma is distinguished from other plasma cell dyscrasias by the abnormal proliferation of plasma cells in a single location, in either bones or soft tissues. It is important to note that extramedullary plasmacytomas (EP) are often seen in patients with MM; however, these lesions are not necessarily solitary and widespread. The symptoms associated with widespread proliferation of abnormal plasma cells are often greater, compared to symptoms seen due to a single lesion [1–7].

Plasmacytoma can be further classified as either solitary plasmacytoma of bone (SPB) or solitary extramedullary plasmacytoma (SEP or EMP). This is dependent on whether the solitary lesion occurs in bones or soft tissues, respectively [1–4]. SPB is more common, accounting for about 70% of all plasmacytomas, while SEP accounts for the remaining 30% [8]. SEP was first described by Schridde in 1905 and can occur in different parts of the body, such as the nasal cavity, sinuses, throat, lungs, or other soft tissues as a solitary lesion [9]. Almost 80% of SEPs are localized in the head and neck region [10].

Plasma cell dyscrasias vary in their presentation; some have widespread symptoms, while others do not. Clinical history and physical examination can be useful, along with laboratory studies to come to a diagnosis of many dyscrasias. Plasmacytoma however can be asymptomatic, and symptoms vary depending on the location of the lesion. Plasmacytoma may be discovered incidentally during imaging or tests for other conditions. In symptomatically silent plasmacytomas, the diagnosis of this disease is often delayed. If left undiagnosed or untreated, plasmacytoma can transform to multiple myeloma or other plasma cell dyscrasias. Despite its rarity, due to the transformation, potential plasmacytoma can cause significant morbidity and mortality in affected patients. Therefore, early diagnosis and prompt treatment are crucial for the management of plasmacytoma [1–4].

The mean age for patients with plasmacytoma varies depending on the specific type of plasmacytoma. Generally, plasmacytoma is more common in older adults, with a median age of onset in the sixth or seventh decade of life. Solitary plasmacytoma of bone (SPB) typically affects individuals in their 50s or 60s, while extramedullary plasmacytoma (EMP), which occurs in soft tissues, usually affects individuals slightly later in life, in their 60s or 70s. However, it is important to note that plasmacytoma can occur at any age, including children and young adults, although rarely [4, 10].

In this case report, we present a case of SEP of the breast in a 79-year-old female patient who was referred to our clinic for evaluation and treatment. Plasmacytomas make up less than 1% of breast neoplasms. Breast plasmacytoma (BP) is an extremely rare plasma cell tumor as the majority of reported cases occur as a result of the disseminated MM. According to the literature, BP accounted for roughly 1.5% of all plasmacytomas; of which, only 15% of these instances were classified as primary BP. The remaining 85% of cases were secondary to MM [6, 11]. Through this case, we hope to provide insight into the diagnosis, treatment, and prognosis of this rare and intriguing type of cancer. By raising awareness and understanding of plasmacytoma, we aim to improve outcomes and quality of life for patients affected by this disease.

## 2. Case Presentation

The patient was a 79-year-old female originally from Honduras. She was referred to our clinic from dermatology due to a complaint of a 0.5 centimeter erythematous, flat, painless lesion on her right breast for 3 years. The patient denied any changes in size to the lesion over time and noted that it never itched. The patient had a past medical history of hypertension and hyperlipidemia controlled with amlodipine and atorvastatin. The patient's family history was positive for colon cancer and ovarian cancer in her older sister and nonrenal cell cancer of the kidney in her younger sister. The patient had no history of prior surgeries.

On physical examination, there was no tenderness to palpation over the right breast and no lump was felt in the right breast. An erythematous lesion was seen on her right breast. The lesion was similar to that seen in a case from On et al.'s study [12]. There were no palpable lymph nodes or other significant findings on examination. Initial laboratory investigations were unremarkable, with no abnormalities in the complete blood count or serum comprehensive metabolic panel. A biopsy was taken at her dermatologist's office, and the dermatopathology report showed the specimen was a plasmacytoma. A subsequent MRI scan showed no additional lesions in the soft tissues or bones, consistent with a solitary plasmacytoma. Bone marrow biopsy was normal. Karyotype showed a normal female karyotype. Fluorescent in situ hybridization (FISH) analysis showed no abnormalities on chromosomes 5, 9, 15, 1p32, 1q21, 13q−/−13, 14q32, 17p13.1, and 17q11. Serum protein electrophoresis (SPEP) showed a normal electrophoretic pattern, a normal gamma globulin level (1.2 g/dL), and a normal beta 2 microglobulin (0.4 g/dL). 24-hour urine protein electrophoresis (UPEP) also showed a normal electrophoretic pattern, a normal gamma globulin level (1.2 g/dL), and a normal beta 2 microglobulin (0.4 g/dL). No monoclonal proteins were present in the urine; however, free kappa light chains were elevated (29.4 mg/L). Lambda light chains were within normal limits (13.3 mg/L). The free light chain ratio was normal (2.21). The patient was diagnosed with stage I tissue plasmacytoma/SEP. Surgery was consulted, and the lesion was removed. Histopathological examination revealed a plasmacytoma. Immunohistochemical staining was positive for CD38, CD138, and kappa light chain. The patient recovered from surgery without complications. Follow-up MRI studies showed postoperative changes at the site of the lesion, with no evidence of disease progression or metastasis. The risk of disease progression was stratified and determined to be low. The patient was counseled on the possibility of disease progression despite low probability. She was informed of the need for regular follow-up to monitor any signs of disease progression.

## 3. Discussion

EMP is less common and less studied compared to SPB [1–4]. The gold standard for diagnosis of SEP is through histological and/or immunohistological analysis of a soft tissue lesional biopsy [8]. Biopsy will show infiltration of monoclonal cells in the affected tissue. Plasma cells in solitary plasmacytoma will be positive for CD138, CD38, and show light chain restriction (stain positive for either kappa or lambda but not both). Beyond biopsy, to be classified as SEP, the following must also apply: normal bone marrow with no evidence of clonal plasma cells, normal skeletal survey and normal MRI (except for at the primary solitary lesion), and absence of end-organ damage such as hypercalcemia, renal insufficiency, anemia, or bone lesions (CRAB) that can be attributed to a plasma cell proliferative disorder [3, 5, 8, 10, 13, 14].

It is important to distinguish SEP from other plasma cell dyscrasias to establish a prognosis and treatment plan. Doing so involves multiple diagnostic techniques including imagining, genetic sequencing, and analyzing the blood, serum, urine, and bone marrow for abnormalities in cells and proteins [8, 15, 16].

### 3.1. Serum and Urine Analysis

**Figure 1:**
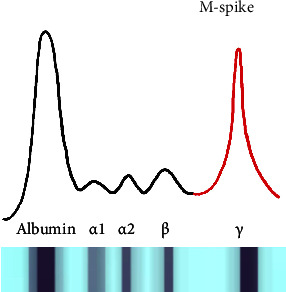
An example of protein electrophoresis seen in a patient with a monoclonal gammopathy. In addition to the normal tall spike for albumin, there is another tall spike. The red line in the gamma region of the graph indicates this abnormal spike. Electrophoresis is useful to determine how much monoclonal protein there is, but not the type. Serum immunofixation electrophoresis (SIFE) is used to determine the isotype of the M-protein. It can further identify the type of light chains (*κ* or *λ*) and heavy chains (*γ*, *α*, *μ*, *δ*, or *ε*) that make up the immunoglobulin.

While the bone marrow and skeletal survey must be normal, patients with SEP may have small amounts of monoclonal protein in the serum or urine. Testing is done using SPEP, 24-hour UPEP, serum immunofixation electrophoresis (SIFE), and free light chain (FLC) ratio [8, 15, 17, 18]. The International Myeloma Working Group classifies multiple different plasma cell dyscrasias with specific criteria along a spectrum [3]. Understanding the immune system is essential when interpreting the laboratory findings in all plasma cell dyscrasias. Plasma cells are immune cells that are further differentiated by B-lymphocytes. They function to produce monoclonal immunoglobulins (antibodies). These are part of the humoral immunity used to fight infections, and also seen in autoimmune diseases. The immunoglobulins secreted by plasma cells are made of proteins. Antibodies have heavy chains (defined by IgG, IgA, and IgM) and light chains (defined by kappa or lambda). Normally, your body will have many different immunoglobulins being produced by different plasma cells. In plasma cell dyscrasias, you will observe abnormal production of plasma cells that have undergone a mutation. These cells secrete a specific type of heavy and/or light chains in abundance (monoclonal protein). This monoclonal protein is seen in abundance on SPEP and UPEP. SPEP is a test that separates proteins from the serum on their electrical charges. Protein electrophoresis is useful for finding deviations in the normal protein fractions found in the serum. Deviations from this normal are seen in conditions such as acute inflammation, alpha-1-antitrypsin deficiency, liver cirrhosis, nephrotic syndrome, and plasma cell dyscrasias, amongst other conditions. In plasma cell dyscrasias, SPEP will show a “monoclonal spike” or “M-spike” in the gamma region on electrophoresis, and examples can be seen in Figure 1. SIFE can be used to obtain more specific information about the type of M-protein present. Obtaining the FLC ratio is useful since well, as monoclonal plasma cells will secrete either kappa or lambda light chains, but not both kinds of light chains. This can cause an abnormal FLC ratio [1–4, 8, 15, 18–21]. Abnormalities in this ratio are a powerful predictor of the risk of progression to multiple myeloma in patients with plasmacytoma. Monitoring for changes in protein electrophoresis can alert progression to MM. In addition, complete disappearance of monoclonal protein following therapy is associated with a low risk of progression to myeloma [15, 19–22].

### 3.2. Bone Marrow Evaluation

Bone marrow biopsy in patients with SEP must show no monoclonal plasma cells. In patients with a bone marrow biopsy that shows a few monoclonal plasma cells (<10%), these patients are further classified as having SEP with minimal bone marrow involvement. More than 10% plasma cells in the bone marrow is a major criterion suggestive of MM [18]. Patients that have SEP with minimal bone marrow involvement are treated similarly to SEP without marrow involvement; however, bone marrow involvement is a risk for transformation into MM [1, 8]. The article by Zou et al. summarizes the histologic features of extramedullary plasmacytomas [23]. While changes seen on bone marrow biopsy are also indicative of disease progression, bone marrow biopsy is invasive and not used for regular monitoring of disease transformation. Repeat bone marrow biopsy may be considered if clinical and laboratory findings are suggestive of disease progression to MM [20].

### 3.3. Imaging

Criteria for SEP are a normal skeletal survey and MRI/CT of the spine and pelvis with no lesions. This excludes the primary solitary lesion [1, 4]. Full body fluorine-18-labeled fluorodeoxyglucose positron emission tomography/computed tomography (18F-FDG PET/CT) scan is used to detect other lesions elsewhere in the body. Soft tissue plasmacytomas can arise from a variety of locations, including muscles, subcutaneous tissue, and viscera, and are often accompanied by nonspecific symptoms such as pain, swelling, or weakness. Imaging findings are nonspecific; plasmacytoma can mimic other soft tissue tumors such as sarcoma or lymphoma in imaging studies. Therefore, a careful histopathological evaluation is essential to differentiate between these entities. Findings are compatible with solid tumors; masses are isointense on T1-weighted images and iso- to hyperintense on T2-weighted images relative to muscles and white matter with variable enhancement. Large tumors may show necrosis and destruction, infiltration, or encasement of adjacent structures. Diagnosis with imagining alone is not feasible as SEP lacks specific radiological and clinical features. When monitoring for disease progression, the imaging modality originally used is preferred to be repeated at follow-up [1, 2, 4, 11, 13, 23–26].

### 3.4. FISH

FISH testing provides important information, as there are high-risk chromosomal changes associated with progression to MM. Testing for these changes is important for the prognosis and treatment. Important chromosomal changes that are associated with a worse prognosis are t (14; 16), t (4; 14), 17p deletion, and 1q amplification. The patient with SEP of the breast had no high-risk genetic mutations. This put her in a lower risk category compared to those who exhibit chromosomal changes. Patients with high-risk chromosomal abnormalities should be monitored more closely as they have a higher risk of progression to MM [15].

### 3.5. Differential Diagnosis

While most breast lesions are due to fibroadenomas with an estimated 10% of the world's female population suffering from fibroadenoma once in a lifetime. A thorough work-up of any breast lesion should include other possible benign and malignant lesions on the differential. It is important to rule out other possible causes including benign fibrocystic changes of the breast, cyst, intraductal papilloma, lipoma, mastitis, phyllodes tumor, fibroma, schwannoma, myxoma, lymphoma, plasma cell neoplasms, Paget's disease, ductal carcinoma in situ, lobular carcinoma in situ, medullary carcinoma, inflammatory breast cancer, invasive ductal carcinoma, and invasive lobular carcinoma amongst other conditions. Clinical presentation, radiographic findings, and biopsy are used in conjunction to come to a definitive diagnosis. While primary plasmacytoma is not a common breast lesion, we report it is a possibility [10, 26, 27].

### 3.6. Treatment and Follow-Up

The treatment of plasmacytoma depends on the location and extent of the tumor. In localized disease, surgical and radiation therapy is the primary treatment modality, whereas in more advanced disease, chemotherapy or stem cell transplantation may be required. While some plasma cell dyscrasias require medical interventions including radiotherapy and chemotherapy [8, 16, 18, 22]. Others may only require follow-up after an initial intervention, such as surgery. The SEP of the breast described in this case fits into the latter category. The lesion was located in the breast and was easily excisable. No abnormalities were observed on follow-up imaging. For all patients with plasmacytoma, it is mandatory to perform a risk stratification to identify any patient at high risk of transformation. Multiple authors have suggested ways to stratify the risk of progression to MM from other plasma cell dyscrasias. However, data to stratify disease outcomes specifically for SEP are limited [15, 21, 28–31].

The prognosis for soft tissue plasmacytoma is generally favorable; however, the 5-year survival rate ranges from 40 to 80%. This leaves a significant amount of uncertainty among both patients and clinicians. The risk of disease progression to multiple myeloma is lower in patients with soft tissue plasmacytoma (10–15%) compared to those with bone plasmacytoma (50–60%) [13]. Even so, close follow-up and monitoring are necessary to detect early signs of disease progression and initiate appropriate treatment. Guidelines and recommendations to monitor the patient and to assess for relapse or progression of this rare disease are lacking. The frequency and extent of follow-up depend on the comfort of both the patient and the physician. Regular follow-up visits with a healthcare provider are important for monitoring the disease and detecting any signs of recurrence or progression. Urine and serum protein electrophoresis with immunofixation, complete blood count, serum creatinine, and serum calcium are commonly monitored at follow-up. In addition, the same imaging modality used prior to therapy should be used periodically thereafter to monitor disease progression. In depth testing including imaging biopsy should be considered if the patient has unexplained symptoms suggestive of MM, or if changes in laboratory studies are observed [4, 8, 10, 15, 18]. While it is important to show the success in this patient and to note her satisfaction with treatment, it is important to add other cases which may present their own unique challenges and may need to be approached differently.

## 4. Conclusion

In conclusion, while plasmacytoma in the bone is more common, soft tissue plasmacytoma can occur and presents unique diagnostic and treatment challenges. Experience in the treatment of this kind of tumor is limited due to the rarity of the disease. A careful histopathological evaluation is crucial to differentiate plasmacytoma from other soft tissue tumors of the breast. Surgical excision with/without radiation therapy is a viable treatment modality for localized disease. Despite the generally favorable prognosis for soft tissue plasmacytoma, close follow-up and monitoring are necessary to detect early signs of disease progression, and to initiate appropriate treatment. While solitary tissue plasmacytomas of the bone are more common and have been more studied, determining the risk of disease progression for SEPs provides its own set of challenges. Due to the rarity of SEP, further investigation is needed to stratify the risk of disease progression more accurately in these patients.

## Data Availability

No data were used to support the findings of this study.
